# Real-Time In Situ Observation of CsPbBr_3_ Perovskite Nanoplatelets Transforming into Nanosheets

**DOI:** 10.1021/acsnano.3c02477

**Published:** 2023-07-05

**Authors:** Aarya Prabhakaran, Zhiya Dang, Rohan Dhall, Fabrizio Camerin, Susana Marín-Aguilar, Balaji Dhanabalan, Andrea Castelli, Rosaria Brescia, Liberato Manna, Marjolein Dijkstra, Milena P. Arciniegas

**Affiliations:** † Istituto Italiano di Tecnologia, Via Morego, 30, 16163 Genoa, Italy; ‡ Dipartimento di Chimica e Chimica Industriale, Università degli Studi di Genova, Via Dodecaneso, 31, 16146 Genova, Italy; § National Center for Electron Microscopy, Molecular Foundry, Lawrence Berkeley National Laboratory, Berkeley, California 94720, United States; ⊥ School of Materials, Shenzhen Campus of Sun Yat-sen University, No. 66, Gongchang Road, Guangming District, Shenzhen, Guangdong 518107, People’s Republic of China; ∥ Soft Condensed Matter, Debye Institute for Nanomaterials Science, Utrecht University, Princetonplein 1, 3584CC Utrecht, The Netherlands

**Keywords:** in situ TEM, in situ heating, shape transformation, perovskite nanoplatelets, self-assembly

## Abstract

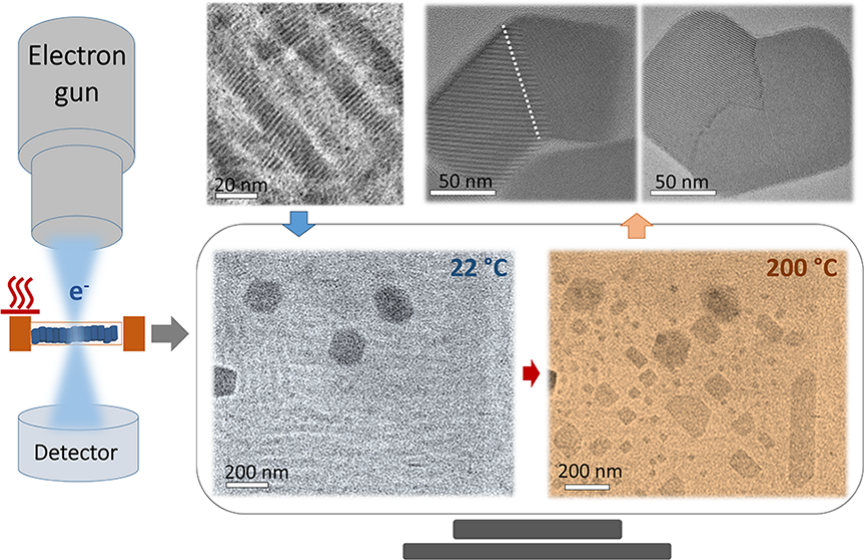

The manipulation of nano-objects through heating is an effective strategy for inducing structural modifications and therefore changing the optoelectronic properties of semiconducting materials. Despite its potential, the underlying mechanism of the structural transformations remains elusive, largely due to the challenges associated with their in situ observations. To address these issues, we synthesize temperature-sensitive CsPbBr_3_ perovskite nanoplatelets and investigate their structural evolution at the nanoscale using in situ heating transmission electron microscopy. We observe the morphological changes that start from the self-assembly of the nanoplatelets into ribbons on a substrate. We identify several paths of merging nanoplates within ribbons that ultimately lead to the formation of nanosheets dispersed randomly on the substrate. These observations are supported by molecular dynamics simulations. We correlate the various paths for merging to the random orientation of the initial ribbons along with the ligand mobility (especially from the edges of the nanoplatelets). This leads to the preferential growth of individual nanosheets and the merging of neighboring ones. These processes enable the creation of structures with tunable emission, ranging from blue to green, all from a single material. Our real-time observations of the transformation of perovskite 2D nanocrystals reveal a route to achieve large-area nanosheets by controlling the initial orientation of the self-assembled objects with potential for large-scale applications.

Two-dimensional (2D) metal-halide perovskite colloidal nanocrystals (NCs) hold great promise for a variety of exciting applications, including X-ray scintillation,^1,2^ photodetection,^3^ lasing,^4^ and optical logic circuits.^5^ The shape and size diversity of such low-dimensional structures enables the preparation of 2D nanomaterials with a thickness of only a few unit cells using diverse synthesis processes, demonstrating tunable quantum confinement.^6−8^ One important characteristic of these NCs is their ability to transform in shape and size by applying post-synthesis treatments,^9^ such as changes in solvent polarity,^10^ temperature,^11−13^ and light illumination.^14,15^ Moreover, regulating chemical or physical conditions allows adjustment of the transformation rates, accelerating a spontaneous process that, when left to its own, can take weeks.^12,16^

Precise heating is a powerful approach for activating single NCs and promoting their fast aggregation and merging to generate structures with optoelectronic properties distinct from those of the original NCs. Through heating, the introduced structural defects, such as grain boundaries^17^ and Ruddlesden–Popper (RP) faults,^18^ can also be modulated with further effects on the optoelectronic properties. These events can occur in both solution^10,12^ and dried films,^15,19^ and the presence of the solvent favors NC rotation and attachment in solution.^9,20^ In films, neighboring NCs can undergo stitching, irrespective of their relative orientation due in part to their reduced mobility, resulting in large structures with random shapes.^19^ Also, the NCs are terminated with ligands, and a key path for their shape transformation consists of ligand detachment and/or relocation,^19^ particularly when suspended in a solvent.

To date, the monitoring of shape transformation of NCs is performed by ex situ transmission electron microscopy (TEM) using the “quench-and-look” strategy.^12,19,21^ While this strategy has provided important insights into the transformed structures, it does not yield details on the dynamics of the transformation, which would enable one to understand how the merging occurs and suggest potential strategies to control the shape of the transformed structures.

A particularly useful “building block” for the shape transformation of NCs is a perovskite nanoplatelet (NPL),^22^ which can transform into various structures such as nanowires,^12^ nanosheets,^10,12^ nanobelts,^15^ nanorods,^23^ and nanocubes.^10^ In this context, CsPbBr_3_ perovskite NPLs are a very appealing system, as they transform over time into extended nanosheets^12^ starting from self-assembled structures.^24^ This transformation occurs spontaneously and enables structural changes that lead to modifications of optoelectronic properties, evolving from blue-emitting, self-assembled NPLs to green-emitting nanosheets.

In this work, we present the direct observation of the structural transformations of self-assembled CsPbBr_3_ NPLs into nanosheets occurring on a short time scale through in situ heating TEM performed on dried films prepared via drop-casting. Three distinct growth pathways are observed in these experiments, which lend mechanistic insights into the formation of nanosheets from the original NPLs. Accompanying molecular dynamics simulations of coarse-grained NPLs show us that there exist different regimes of the growth of these NCs characterized by distinct growth rates. While these experiments allow us to identify the different stages of the growth mechanisms, the accompanying interactions of the electron beam with both the ligands^19^ and the perovskite NCs introduce significant challenges that require the use of low-dose imaging. We also performed these observations on dried films, which adds another challenge to the experiments. To ensure that these experiments are meaningful, we compare the results to ex situ heating conditions and investigate the optoelectronic properties of the structures obtained at different stages. Time-resolved photoluminescence (PL) analysis shows that temperature-driven transformations of NPLs create significantly longer decay lifetimes, which we can relate in our study to the observed structural changes.

## Results and Discussion

### Sample Details

**Figure 1:**
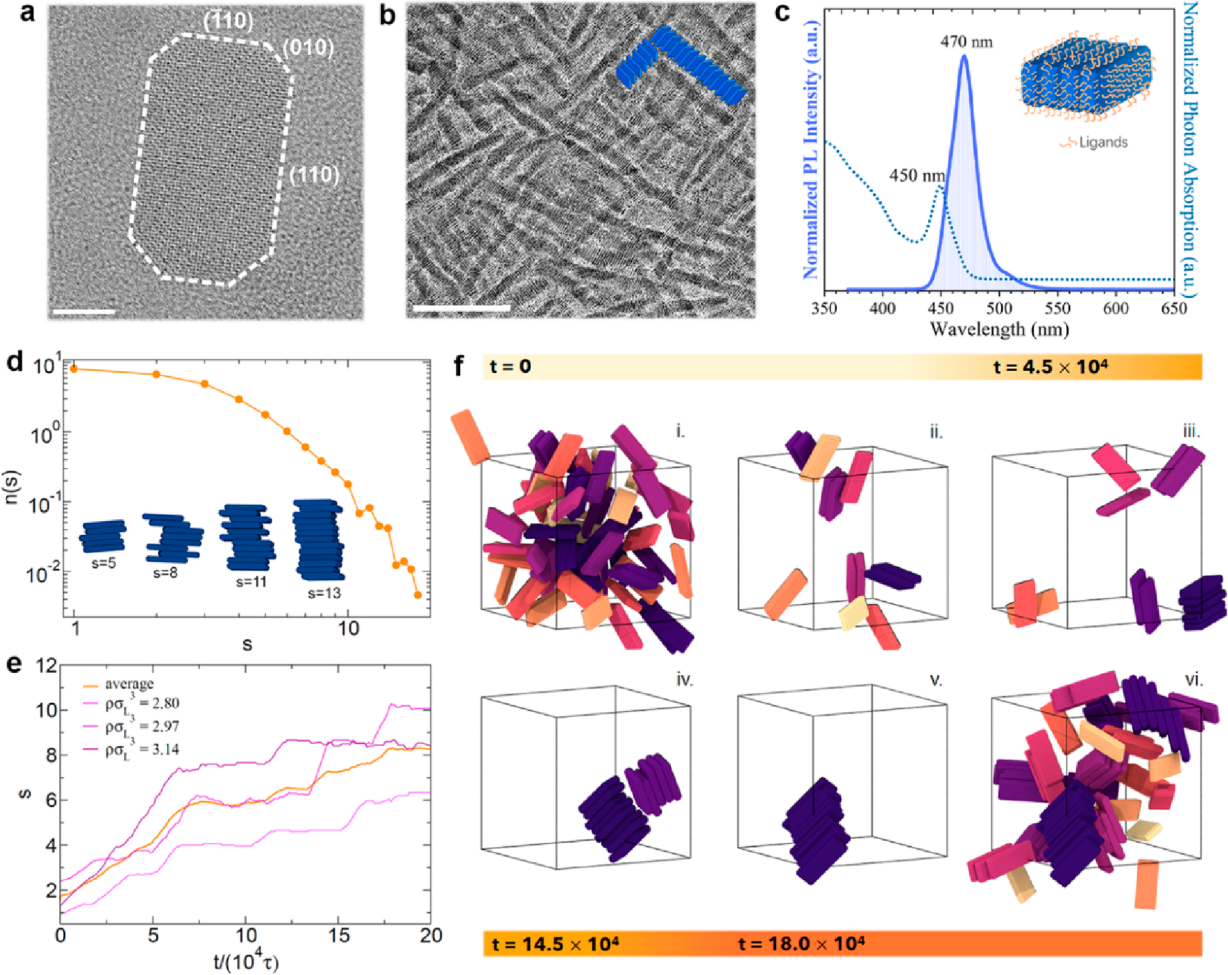
Structural details. (a) High-resolution TEM image of a NPL laying with its basal plane parallel to the C support film. The indexing is based on the orthorhombic CsPbBr_3_ phase (ICSD 97851).^15^ Scale bar: 5 nm. (b) Bright-field TEM image showing the self-assembly of NPLs into ribbonlike structures, as illustrated in the embedded sketch. Scale bar: 200 nm. (c) Optical absorption (dotted line), and PL spectrum (solid line) of ribbons dispersed in toluene. Inset: a 3D cartoon of a short ribbon illustrating the ligand distribution on the NPL surfaces. (d) Ribbon size distribution *n* (s) as a function of the ribbon size *s*, as obtained from molecular dynamics simulations. Data are averaged over nine independent configurations at a density of NPLs of approximately ρσ_*L*_^3^ ≈ 3 with σ_*L*_ being the length of a NPL. The inset shows simulation snapshots of representative ribbons formed by different numbers of NPLs. (e) Size of the largest identified ribbon *s* as a function of simulation time *t* in units of τ, with τ being the unit of time in the simulations. (f) Typical configurations observed in the initial (i) and final (vi) simulations stages. In panels (ii)–(v), simulation snapshots show the formation of the largest identified ribbon over time. Note that panels (i), (ii) and (v), (vi) correspond to the same simulation time steps. NPLs with different colors belong to different ribbons and unwrapped coordinates are used.

We examine aliquots of 15 μL of CsPbBr_3_ NPLs dispersed in toluene and drop-cast on carbon-coated Cu TEM grids. The NPLs were synthesized following our previously reported synthesis.^12^ NPLs can be depicted by brick-like shapes with truncated corners (Figure 1a). The NPLs have average dimensions of 21 nm in length (*l*) and 8 nm in width (*w*), with a thickness (*t*) of approximately 3–4 nm. These structures self-assemble into face-to-face stacks, referred to as ribbons in this study, as shown in Figure 1b, which indicates their nearly monodisperse size. The inter-NPL distance within ribbons is about 3 nm (Figure S1). Each facet of a NPL is passivated by Cs- and Pb-oleate and oleylammonium-Br.^12^ We infer a higher number of ligands passivating the basal planes compared to the edge facets, as they have a larger surface area. The pristine solution of NPLs shows a single and strong absorption peak at 450 nm and a PL peak at around 470 nm, with a full-width at half-maximum (FWHM) of approximately 24 nm (Figure 1c), confirming the formation of ribbons dispersed in the toluene solution.^12^ A minor emission at 510 nm is also observed, likely arising from larger structures compared to the ribbons. Despite the efficient energy transfer from NPLs in the ribbons to these large structures, the peak at 510 nm exhibits very low intensity, suggesting that an ultrasmall population of objects with a different morphology developed in solution during the time between the synthesis and the TEM experiments.

To provide additional evidence of ribbon formation under 3D conditions, that is, in solution, we conducted molecular dynamics simulations. Since our focus is on mesoscopic length scales, we represent each NPL as a bead-tessellated object with a shape and aspect ratio that approximate the experimental dimensions. To capture the effect of ligands on the NPL surfaces, we incorporate an attractive interaction between different NPLs in a coarse-grained manner while treating the solvent implicitly. This modeling allows us to include the essential elements required for studying the key steps driving the self-assembly and the transformation mechanisms without accounting for details about the ligands or specific microscopic processes that occur in our experiments. Incorporating such detailed features in the simulation would also make it computationally infeasible. By employing this coarse-grained model instead, we gain insights into the formation of ribbons under three-dimensional conditions (as it occurs in solution), complementing the experimental observations. Further details on the modeling and simulations can be found in the Experimental Section.

We start by analyzing the size distribution of the ribbons that are formed from the individual NPLs in simulations. We consider a NPL as part of a ribbon when the distance between the basal planes of different NPLs corresponds to the minimum of the attractive interaction potential and when the NPLs have the same orientation along the longest lateral side. The average size distribution of the resulting clusters (ribbons) at the end of nine independent simulation runs is displayed in Figure 1d, which shows a wide range of ribbon sizes. While single NPLs or small ribbons are still present, we also observe the formation of large ribbons consisting of ten or more NPLs. Similar to the experimental observations, the simulations show that the NPLs self-assemble primarily with their basal planes (large facet) aligned to each other, because the ligands located at the basal planes have the strongest attractions due to the largest surface area compared to the other (edge) facets. We show some representative snapshots in the inset of Figure 1d for different ribbon sizes. The configurations of the NPLs in the simulations are indeed qualitatively comparable to the ribbons observed in Figure 1b of the experiments with a strong preference for retaining a linear conformation and a slight offset in the position of two consecutive NPLs.

Simulations also allowed us to follow the formation of ribbons as a function of time. Specifically, we focus on the largest identified ribbon in each run and study its size during the course of the simulation. Figure 1e shows the results for three different NPL densities together with their average values from three independent runs for each density. In all cases, we observe three distinct regimes in the formation of ribbons. The first regime, up to ≈7 × 10^4^τ, is characterized by a steady growth in the size of the ribbon. This is followed by a regime in which the size remains roughly constant before a final growth occurs in the last part of the simulation. Figure 1e shows that the final average size of the ribbons is around 8 NPLs, but can be as low as 6 NPLs and as high as 10 NPLs for the range of densities investigated in these simulations. Irrespective of the density of NPLs, the final size of the aggregate ranges between 6 and 12 NPLs. At decreased NPL densities, longer assembly times are expected for observing the formation of longer structures. The growth of a ribbon is reported in Figure 1f, where we show typical configurations over the course of a simulation. In all panels of Figure 1f, different colors represent different ribbons. For visual clarity, the coordinates of the NPLs displayed in Figure 1f are unwrapped, meaning that they are presented as they would appear if they had not been wrapped back into the periodic simulation box. Note that configurations (i), (ii) and (v), (vi) in Figure 1f correspond to the same simulation time steps, with (i) and (vi) showing the initial and final states, respectively. For the sake of clarity, only the NPLs which eventually aggregate into the longest ribbon are shown in frames (ii)–(v), but frames (i) and (vi) do show the other NPLs in the simulation box, which also undergo aggregation. As can be observed from the figure, growth proceeds through the gradual merging of individual NPLs until two intermediate-sized ribbons are formed (panel (iv)). In the final step, these two objects merge into a ribbon with a size of *s* = 13 NPLs. Other ribbons of different sizes can also be observed in configuration (vi) of Figure 1f. This also highlights two distinct assembly pathways: the addition of a single NPL to a ribbon and the attachment of two separate ribbons. While the first pathway leads to a gradually increasing size of the formed ribbon over time, the second pathway leads to sudden jumps in the length of the assembled ribbon, as shown in Figure 1e. In these jumps, the size of the ribbon grows by more than 1 NPL within a few simulation time steps, and these jumps are more common in the simulations where the original density of NPLs is the highest. However, both of these assembly pathways pertain to the attachment of NPLs along their basal planes.

In summary, we consistently observe both in experiments and in molecular dynamics simulations of coarse-grained NPLs that the starting sample is primarily composed of NPLs with their basal planes self-aligned to form ribbons, as depicted in Figure 1c. This arrangement is likely due to the stronger interactions of ligands at the basal planes (larger facets) compared to the edges,^25^ as typically observed in similar 2D materials.^26,27^

### In Situ TEM Heating Observations

We performed in situ TEM heating experiments on fully dried samples on a substrate prepared by drop casting an aliquot of 15 μL on a C coated Cu TEM grid. We used an in situ heating holder (Gatan 652) compatible with the Thermo Fisher Themis TEM to carry out detailed observations (see details in the Experimental Section). The samples were imaged in TEM mode at a low magnification of 6300× to avoid large misalignment due to thermal drift, and movies were recorded on a Ceta Camera (4k × 4k) capable of recording at high frame rates necessary to capture dynamic events. In the initial examination of the samples at room temperature (22 °C), we focused on areas with different ribbon densities, which we labeled as R1 and R2 to indicate low and high ribbon concentrations, respectively (Figures S2 and S3). We observed that R1 areas, such as those shown in Figure S2, contain ribbons with a length ranging from 30 to 190 nm. In contrast, R2 areas are rich in ribbons ranging from 60 to 230 nm, which appear well-packed in a parallel arrangement with relatively closer distances (Figures S1 and S3). The large objects (dark contrast) observed in the TEM images collected at 22 °C correspond to zero-dimensional Cs_4_PbBr_6_ NCs formed during the storage period before the experiments,^12^ which are used here as landmarks for drift correction and to track the change in the different areas and facilitate frame alignment. Next, the temperature was increased from 22 to 100 °C in 5 min, and the samples were kept at this temperature for 35 min to match experimental conditions with ex situ heating and PL experiments performed from fresh solutions and presented in Figure S4. Finally, we increased the temperature to 200 °C. The heating ramps of the complete experiments are illustrated in Figure S5. All of the heating ramps were performed with the electron beam switched off to minimize possible effects from electron irradiation. Each selected region was then monitored at the target temperatures.

**Figure 2:**
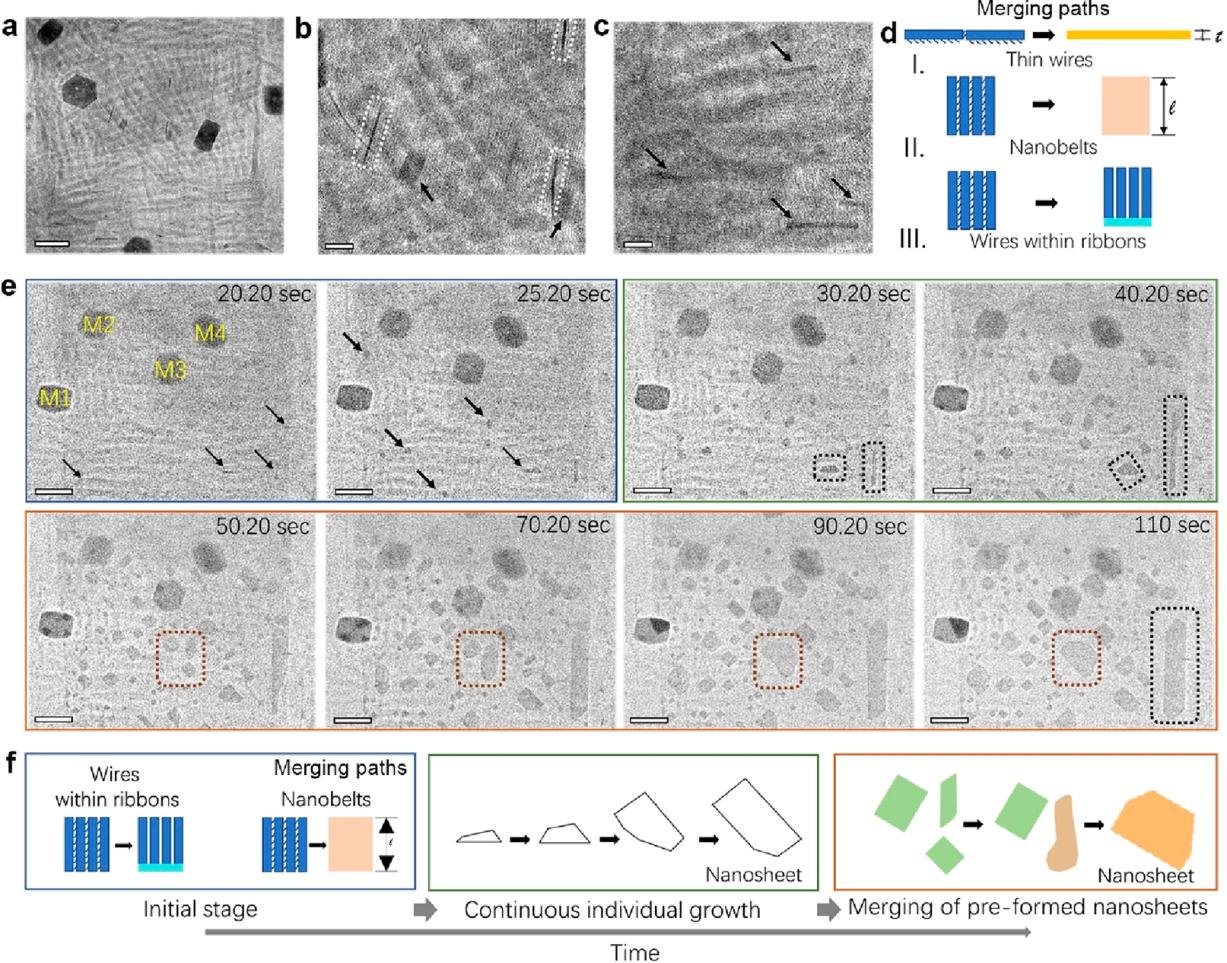
Stages of the NPL transformation induced by heating. (a–c) Bright-field TEM images showing the initial merging of NPLs observed in R2 areas at 100 °C after 35 min. Magnified views of panel (a) are displayed in panels (b) and (c). Scale bar in (a): 200 nm; (b, c): 50 nm. Representative *thin wires* are framed in white in panel (b); *nanobelt*s and *wires within ribbons* are indicated with arrows in panel (c). (d) Sketch illustrating the different merging paths of NPLs: I. Merging of neighboring NPLs through the NPL’s edges (*thin wires*); II. Merging of NPLs, which are part of the same ribbon, through their basal planes (*nanobelts*); and III. Merging of the end portion of NPLs within the same ribbons to form *wires within ribbons*. (e) Selected frames from Movie S2 showing the evolution of the ribbons toward nanosheets at a high temperature (200 °C), starting from 20.20 to 110 s. M1–M4 indicate the landmarks used to align the real-time images. The arrows indicate some of the *wires within ribbons* observed at 20.20 s and *nanobelts* at 25.20 s. The black dashed frames highlight the growth of single objects in different orientations and the brown ones the merging of close nanosheets. Scale bars: 200 nm. (f) Sketches showing the different stages of the transformation in time.

We performed our first observation after 35 min at 100 °C and noticed slight morphological changes in ribbon-rich areas (R2) where we identified three types of objects (Figure 2a–c), which are randomly dispersed in the field of view (FOV) and are not formed by single NPLs, but originate from partially or fully merged NPLs:^12^ (i) *thin wires* of ca. 3–4 nm in thickness (white-framed objects in Figure 2b); (ii) *nanobelts* with a width of ca. 21 nm (indicated by arrows in Figure 2b); and (iii) *wires within ribbons* that refer to thin wires observed down the long side of a ribbon (Figure 2c). The dimensions of objects (i) and (ii) match well with the thickness and width of the initial NPLs. Based on their dimensions, we infer three different merging pathways, as illustrated in Figure 2d: *thin wires* originate from the merging of NPLs through their edge facets (Figure 2d, pathway I) and *nanobelts* are formed by merging ribbon’s NPLs through their basal planes (large facets), see Figure 2d, pathway II. Note that in the nanobelts the basal plane ligands have been desorbed, while a ribbon still retains the original ligands on the basal planes, creating a superlattice-like morphology. The *wires within ribbons* in Figure 2d, pathway III, appear to be generated by the initial merging of the ribbon’s NPLs through their end portion, which could explain why they are located along the ribbon’s side.

Unlike in the liquid phase, desorption of ligands from NC’s surfaces on a solid substrate does not easily occur. Instead, ligands can rotate with one end still bonded to the NPL. From our experiments we infer that ligands at the edge facets of the NPLs have an advantage in motion compared to those located at the basal planes (large facets), as these surfaces are passivated by a smaller number of ligands,^25,28^ which facilitates ion migration.^29,30^ This can explain the initial preferential growth pathway I observed at such low temperatures, which also favors pathway III. Note that our experiments are performed at temperatures below the potential sublimation of ligands and degradation of NCs. Unbound ligands of oleic acid and oleylamine degrade above 240 °C^31^ and ligand-free CsPbBr_3_ nanocrystals remain stable at temperatures below 417 °C under TEM vacuum conditions.^32^ Given the higher degradation temperature of the nanocrystals compared with that of the ligands, it is expected that the degradation temperature of ligands bound to the surfaces of the nanocrystals will also increase further. Nevertheless, the labile nature of the ligands on the nanocrystal’s surface^33,34^ contributes to their desorption during heating at relatively low temperatures.^35^

In the second part of our experiments, we sought to explore a higher temperature and investigate further the potential transformation of preassembled structures on a substrate to offer a complete view on the mechanism. Note that the inspection of the areas at different magnifications after heating at 100 °C have created regions irradiated with lower and higher electron doses (Figure S6) that resulted in heavier carbon contamination in circular areas (the beam shape).^36^ At 200 °C, we did not observe changes in preirradiated regions. This is attributed to the cross-linking suffered by ligands under both high energy radiation of electrons and X-rays,^19,37^ which hamper further significant motion of ligands from the large facets of the NPLs. While the R1 areas did not show significant changes (Movie S1), we evidenced a series of events in the R2 area (lower cumulative electron dose) that remained active at such a high temperature (Movie S2). Figure 2e displays a series of snapshots extracted from Movie S2 (see magnified snapshots in Movie S3) during the first 110 s of heating at 200 °C when major morphological changes occurred. By tracking the morphology of different objects observed in the FOV, we have established three different stages of the transformation of the dried NPLs on the C-coated Cu grid (Figure 2f): (i) (early stage) the merging of NPLs within ribbons via removal of ligands on the basal planes, leading to growth pathway II and favoring pathway III; (ii) (intermediate stage) the continuous growth of single objects to form defined nanosheets; and (iii) (late stage) the merging of close nanosheets formed in stage ii. From a magnified view of the area extracted from Movie S2 (Movie S3), the early stage of the transformation at a high temperature is confirmed to be characterized by growth pathway II and III (see blue framed panels in Figure 2e). Eventually, the resulting objects attach to form larger and faceted nanosheets in a few seconds, within two size populations: relatively small nanosheets of up to 75 nm in length and nanosheets of up to 100 nm. These nanosheets appear randomly dispersed in the FOV. The black dashed frames in Figure 2e highlight single objects that grow continuously over time. At a late stage, neighboring nanosheets also merge into large ones (follow brown dashed framed objects in Figure 2e), exposing only the low index facets to minimize the surface energy.^38^ The grids used in the heating experiments were inspected 24 h after using a single tilt holder to acquire energy dispersive X-ray spectroscopy (EDS) maps in scanning transmission electron microscopy (STEM) of the final structures. The EDS analysis confirms that the nanosheets retain the chemical composition of the initial perovskite NPLs, see Figure S7 and Table S1.

**Figure 3:**
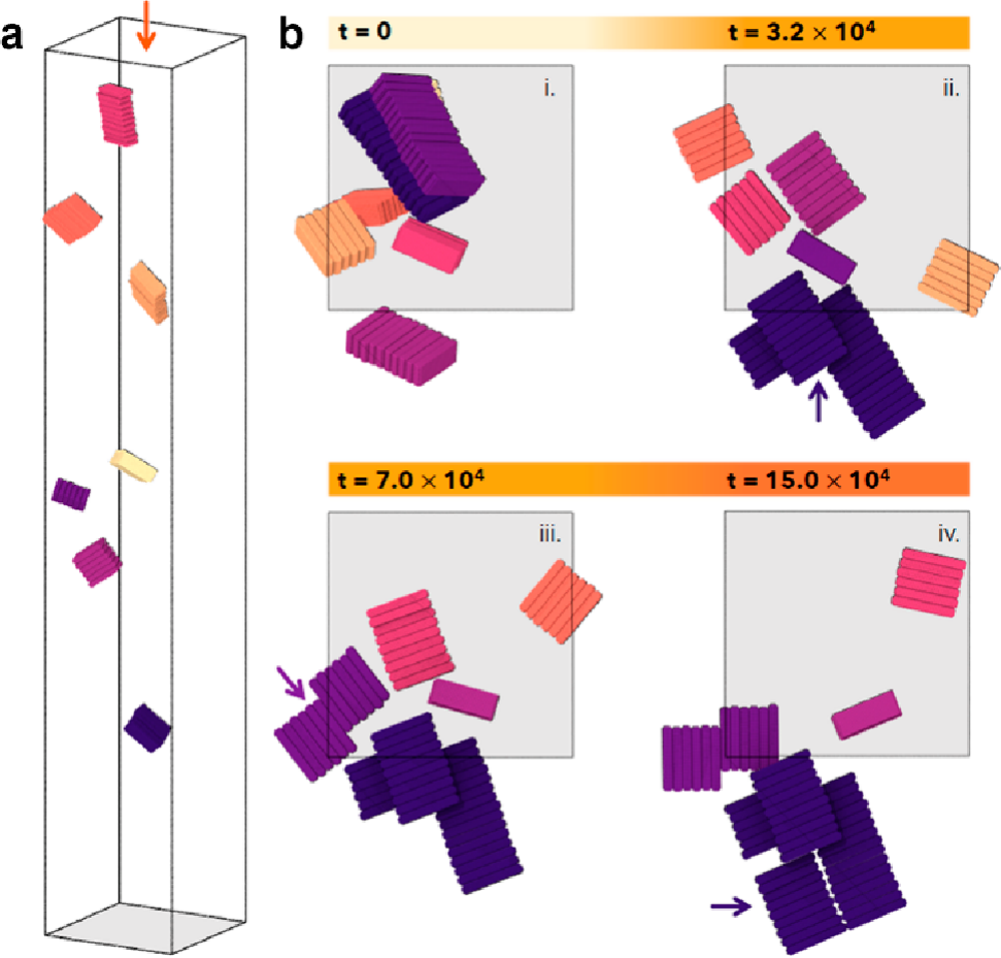
Formation of ribbons and nanosheets on a substrate. (a) Typical configuration showing the initial state of the simulation in which ribbons of different sizes are placed at random positions and with random orientations in an elongated simulation box. (b) Top views of the elongated simulation box (orange arrow in panel (a)) showing the time evolution of the deposition and transformation of the preformed ribbons. The arrows indicate the merging of pre-existing ribbons via the basal plane or the short edges during the course of the simulation. In all panels, different colors relate to different ribbons, and unwrapped coordinates are employed. The gray areas indicate the substrates. Times are reported in units of τ.

For dried films of NPLs on a substrate (two-dimensional conditions), we observe similar transformation pathways in molecular dynamics simulations by starting with a set of ribbons of different sizes and letting them deposit on a substrate, as shown in Figure 3a. In the figure, NPLs belonging to the same ribbon are depicted in the same color. We aim to provide a qualitative description of the steps that lead to the potential formation of nanosheets. For this reason, we do not explicitly consider any effect related to the change in concentration that would typically occur in such dry processes. Additional simulation details are presented in the Experimental Section. Once the ribbons are deposited (Figure 3b), we observe the spontaneous formation of larger structures, indicated by the violet arrow in panel (ii), where two ribbons merge on the short edge and a third one deposits on top of the first two. We also observed a ribbon that is deposited through its largest facet and does not participate in the formation of longer ribbons or more extended structures. At longer times (iii–iv), we observe the formation of extended structures starting from two shorter ones made of six NPLs each (purple arrow in panel (iii)). In this case, the process is similar to the observed transformation, where long nanobelts are formed by merging through the basal planes of the NPLs (growth pathway II). In the last step, as shown in panel (iv), the structure previously formed in panel (iii) is enlarged by connecting another ribbon of intermediate size via its short side (violet arrows in panel (iv)). The structure that is formed in this way can be considered as a precursor of the nanosheets that are observed in experiments, where objects observed in stage (i) merge through various paths, as supported by the simulations. However, the observation of larger structures is restricted by the current modeling limitations, as this would require a higher number of NPLs, increasing the computational costs significantly.

**Figure 4:**
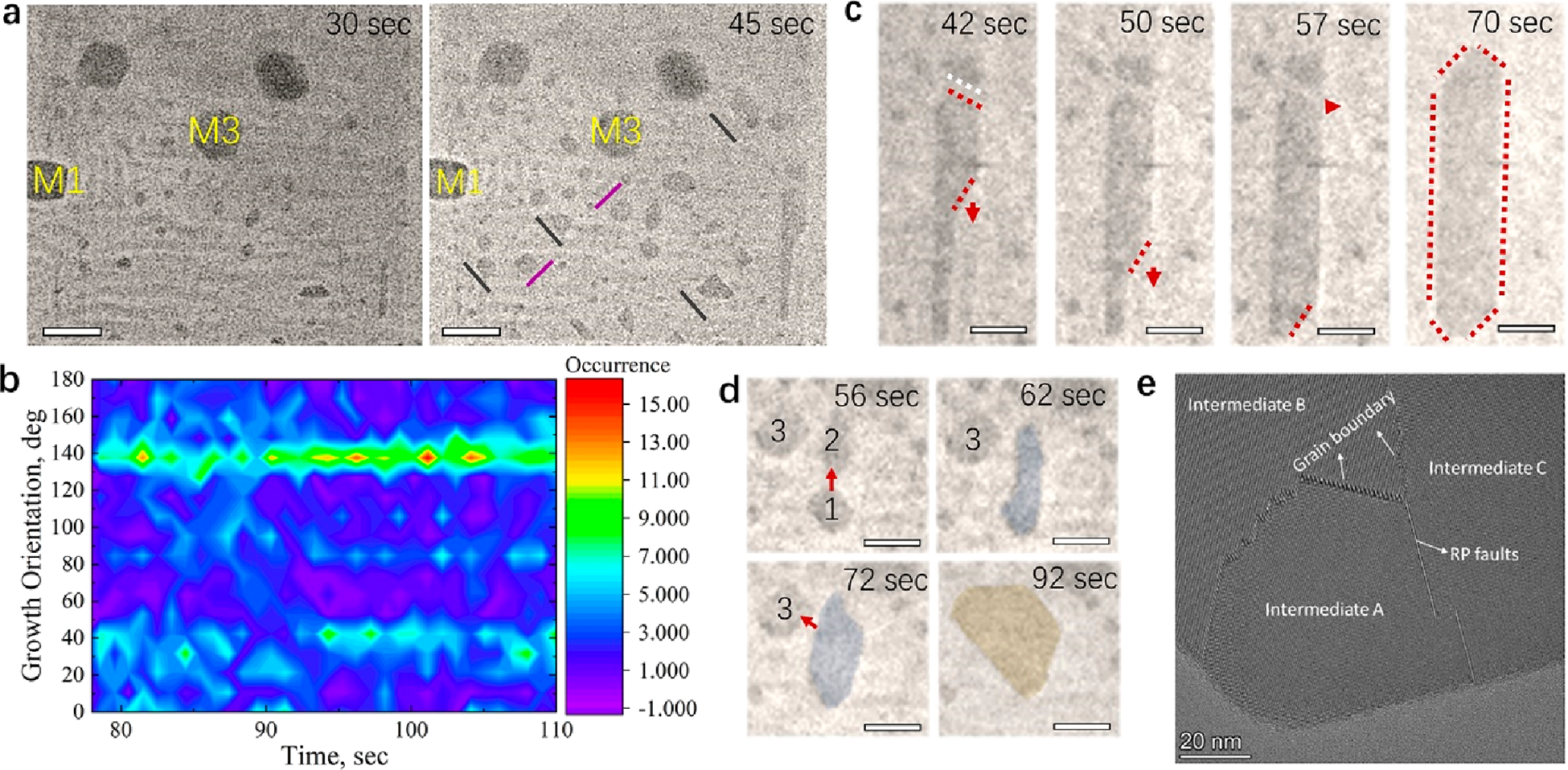
Oriented growth of CsPbBr_3_ nanosheets at high temperature. (a) Bright TEM images extracted from Movie S3 showing the preferential orientation (highlighted with solid lines) of different objects when transforming into nanosheets over short times (up to 45 s) at 200 °C. (b) Orientation mapping of the objects growing in the FOV over time. (c) A series of snapshots showing the formation of a large nanosheet, which shows a vertical growth (90°) with respect to the orientation of the initial ribbons in the region (0°). The dotted white line highlights the tilted facet of a small nanosheet, where the merging occurs. The red arrows indicate the growth orientation observed at different times. Scale bars: 100 nm. (d) Selected frames from Movie S3 showing the merging of three preformed nanosheets over time in the FOV. The red arrows indicate the merging direction. Scale bars: 100 nm. (e) High-resolution TEM image of a final nanosheet highlighting the presence of grain boundaries and Ruddlesden–Popper (RP) planar faults and hinting at the different intermediates merged to form the nanosheet.

To achieve a comprehensive understanding of the transformation of perovskite NPLs to nanosheets on a solid substrate, we then experimentally tracked the growth of single objects in the FOV. We found that there is a preferential orientation that favors the attachment of nearby ribbons through preformed objects, such as wires and nanobelts, or small nanosheets. Some of these initial objects are indicated in Figure 4a.

Interestingly, most of them adopt a 45° or 135° orientation (Figure 4b) with respect to the elongated axis of the initial ribbons from which they originated. Only a very few structures in the FOV start to merge in a fully vertical orientation (90°). Figure 4c shows one of the larger nanosheets that resulted at a high temperature with this configuration. In this case, growth likely occurs through the merging of nearby nanobelts from different ribbons located within a few nanometers and parallel to each other. However, the merging with preformed nanosheets (as the one located at the right-top corner of this object in Figure 4c) remains at 45° or 135°.

We reasoned that the different orientations observed for the growth of the nanosheets are strongly related to the initial path activated by the temperature and the distance between initial ribbons: The majority of the nanosheets emerge from short nanobelts (with ca. 7–10 NPLs) in areas where the initial ribbons appear parallel with respect to each other; the distinctive waving of ribbons (inset in Figure 1d) creates shorter distances, favoring the merging of the initial nanobelt with a section of the closer ribbon through their lateral sides. This indicates that the ligands at the sides of the ribbons remain highly active at a high temperature, and thus they are prone to generate a merging path. The sketch in Figure S8 shows the elucidated ligand detachment/relocation and lattice merging from two neighboring NPLs within a ribbon.^34,39^ We note that, in contrast with the self-assembly in the solution that led to nanosheets with defined rectangular shapes,^12^ the nanosheets formed at the late stage of the transformation on the substrate display random shapes (see example in Figure 4d). We attribute this to the random merging paths originated from the different orientations of the initial ribbons, the changes in their local distribution, and their related distances, as it occurs in nanocubes,^19^ but also due to the reduced mobility of the NPLs in the substrate. Therefore, to achieve control over the final morphology of the transformed NCs, we foresee three strategies: (i) control over the initial NPL density to favor the formation of uniform films from single ribbon layers via solution-based processes;^25,38^ (ii) templating the initial orientation of ribbons into the desired configuration to favor one specific merging path;^40−42^ and (iii) ligand engineering to control the distance between initial ribbons and their orientation.^43,44^

To detail the atomic merging of the objects, we performed a high-resolution TEM analysis of the resulting nanosheets. Contrary to the spontaneous transformation at room temperature and solution where the dominant defects are RP planar faults,^12^ we often observe grain boundaries and, in a few cases, RP planar faults. These defects are located at the interface between neighboring intermediates with continuous perovskite lattices. Our reasoning is that the initial ribbons can easily rearrange their orientation, and thus intermediate objects show a continuous perovskite lattice. During this initial stage, ligands are partially desorbed into the vacuum and partially remain at the nanocrystal surfaces. The removal of ligands along with surface atoms during the ligand desorption process generates vacancies, which help the formation of a perfect perovskite lattice (see the sketch in Figure S8). At this point, intermediate objects are (i) bulkier compared with the initial objects and thus have less mobility, and (ii) very little ligand desorption is involved, and vacancy-mediated merging is lacking. Hence, the merging of intermediates occurs without much adjustment, and thus grain boundaries are commonly observed (see additional high-resolution TEM images in Figure S9). These events take place on a solid substrate and at a higher density of nanocrystals while in a liquid medium and at a lower nanocrystal density, the intermediates could still rotate and merge into large nanosheets with mainly RP planar faults.^12^

**Figure 5:**
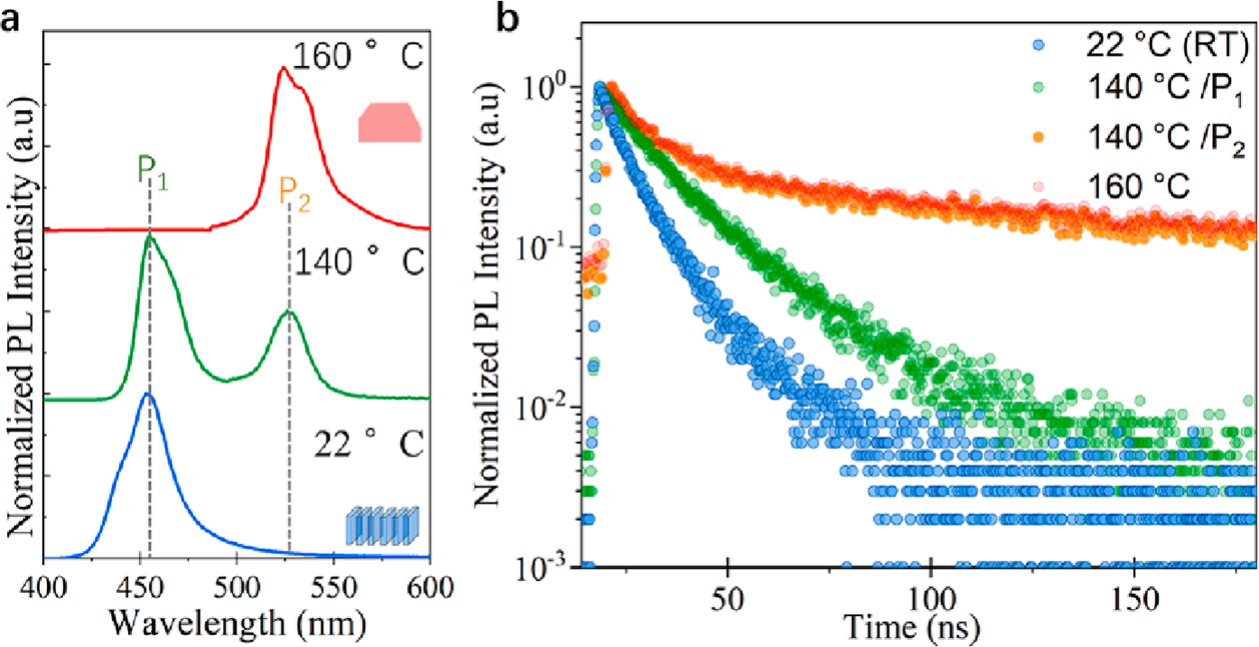
Optical properties of transformed objects in films. (a) PL spectra collected from films prepared at different temperatures. The two emission peaks observed at 140 °C are indicated as P_1_ (455 nm) and P_2_ (526 nm), respectively. The embedded sketches display the ribbons and nanosheet morphology of the objects at the corresponding temperatures. (b) Time-resolved PL decays collected from objects formed in films at different temperatures.

To investigate the effect of the heat-induced transformation on the optoelectronic properties, we performed PL and time-resolved PL analysis on films prepared at different temperatures via drop casting the initial solution containing ribbons (Figure 5).

We observe single emission peaks from films prepared at room temperature and 160 °C, which correspond to the initial and final stages of the transformation (Figure 5a). At 140 °C, we observe two emission peaks (green PL profile in Figure 5a), which are evidence of an intermediate stage of the transformation, as was also observed in solution (Figure S4). Regarding the PL decay times of the structures in films, which were fitted with three-exponential functions (see Table S2), we found that the films of ribbons (blue PL profile in Figure 5b) show a relatively fast τ_2_ component (with ∼65% weight) of ca. 8.0 ns, while the films of fully transformed objects obtained at 160 °C display a more balanced contribution from the slow and fast components (∼30–38% weight), which results in a significantly increased average decay lifetime of ∼170 ns. At 140 °C, the decay lifetimes show a faster decay of τ_2_ = 6.62 ns (∼62% weight) for the blue component (*P*_1_ = 455 nm) while the green component (*P*_2_ = 526 nm) shows similar decay lifetimes as those obtained in films produced at 160 °C. This confirms the presence of fully transformed objects alongside ribbons in the films. An increment in decay lifetime has been observed when transforming similar NPLs deposited on a substrate by UV irradiation^15^ or when directly synthesizing thin nanosheets.^45,46^ This has been explained by either a reduction in the thickness of the nanosheets or a more effective defect passivation. However, the average decay lifetimes in these examples are significantly lower (up to 12 ns) compared with that of the nanosheets produced through heating in our experiments. To elucidate the differences in carrier dynamics between the initial and transformed objects, we performed PL quantum yield (PLQY) measurements and calculated the radiative (*K*_*r*_) and nonradiative (*K*_*nr*_) recombination rates (Table S3). The starting ribbons have a PLQY of 23% while the resulting nanosheets show a drop of up to 8%. Generally, NPLs suffer from a high density of surface defects due to their higher surface-to-volume ratio.^22,47^ The significant contribution of a fast radiative decay lifetime that we observed in the initial NPLs corresponds to the nonradiative recombination channels (Table S3), leading to a lower PLQY compared to bulk NCs. In the case of the nanosheets, we observe a much longer average decay lifetime but no benefit in the PLQY. In comparison with thin NPLs, the thicker transformed nanosheets provide more room for carriers to move and radiatively recombine or trap within surface defects, which explains the slow decay lifetime. However, grain boundaries and other defects (their presence being revealed by the high-resolution TEM images, see Figure 4e and Figure S9) act as bulk defect centers for nonradiative recombination, negatively influencing the PLQY of the resulting nanosheets.^48,49^

## Conclusions

In summary, the in situ monitoring of the transformation of NCs with temperature to capture structural modifications in dried films has been a multiscale challenge. In response, we presented in this work the in situ TEM observations of the heat-induced transformation of CsPbBr_3_ NPLs dried on substrates, which undergo morphological changes to form nanosheets from self-assembled objects, as it was also evidenced by computational analysis. We demonstrated different merging pathways during heating, which lead to large structures with random shapes that display structural defects originated from the merging of preformed neighboring (small) nanosheets. Such objects, obtained from the NPL’s transformation on a substrate, show long decay lifetime that we attribute to an efficient relocation of ligands and, thus, enhanced defect passivation. We envision that well-aligned and packed nanosheets might be formed on substrates through heating by first controlling the deposition of the initial self-assembled objects in solution to create defined patterns with precise control over their orientation.

## Experimental Section

### Synthesis of NPL Ribbons

The synthesis was performed following a previously reported protocol from our group.^12^ Briefly, a solution of 0.145 g of PbBr_2_ in 4 mL of octadecene was prepared in a 20 mL glass vial. To this solution, 1 mL of oleylamine and 1 mL of oleic acid were added. The mixture was heated at 100 **°**C for 20 min with magnetic stirring. In another 8 mL glass vial, 0.325 g of Cs_2_CO_3_ were dissolved in 5 mL of oleic acid at 100 °C for ca. 15 min. With both solutions at room temperature, 0.5 mL of the Cs_2_CO_3_ solution were added to the PbBr_2_ mixture. The mixture was heated at 60 **°**C with magnetic stirring for 30 min. After this time, the solution was cooled down in an ice water bath for 5 min and 2 mL of toluene were added. The solution was centrifuged at 3500 rpm for 10 min and the product was collected and redispersed in 2 mL of toluene, discarding the supernatant.

### In Situ Heating TEM Experiments

The in situ heating experiments were performed in an image corrected Thermo Fisher Scientific Themis instrument operated at 300 kV. The samples were prepared by drop-casting 15 μL of CsPbBr_3_ NPLs in toluene on a carbon-coated Cu-based TEM grid (200 mesh). The grids were loaded into a Gatan 648 double-tilt heating holder and the initial screening of the samples was performed at room temperature (22 °C). Through this process, we identified 2 regions of interest that contained different ribbon densities and images were collected at ∼1000 e/(A^2^·s) electron dose rate. The ramps of temperatures used in the experiments are detailed in Figure S5. All of the ramps were set with the electron beam off to minimize beam-induced effects. The sample was irradiated once the set temperature was reached. Movies were converted to 5 fps for better visualization. All the movies were aligned using as a reference the large zero-dimensional Cs_4_PbBr_6_ NCs observed in the field of view. For STEM-EDS maps, the Br K-edge and Cs and Pb L-edges were used for all of the structures. For pristine NPLs, high-resolution TEM images were acquired on an image-corrected JEOL JEM-2200FS TEM, operated at 200 kV. HRTEM images presented here have been acquired using a direct-electron-detection camera (K2 Summit, Gatan), so as to reduce beam damage, and post-processed by average background subtraction filter.^50^ For these analyses, a diluted solution of NPLs (25 μL in 1 mL of fresh toluene) was prepared and drop-cast onto a double carbon film (ultrathin C on holey C) on a Cu grid. The measurements of ribbons lengths were performed in areas of 500 × 500 nm.

### Optical Characterization

PL and absorbance spectra were collected by using a Varian Cary 5000 UV–vis-NIR spectrophotometer. The time-resolved photoluminescence measurements were carried out at the maxima of the emission peak on an Edinburgh Instruments fluorescence spectrometer (FLS920) with a time correlated single-photon counting (TCSPC) unit coupled to a pulsed diode laser. The samples were excited at 375 nm with a 2 μs pulse repetition period. Measurements from solutions heated at the different temperatures were recorded by diluting 10 μL of fresh toluene. For films, 25 μL of as-synthesized NPLs was drop-cast on 5 × 5 mm Si/SiO_2_ substrates and heated on a hot plate at the respective temperatures for 30 min. The PLQY values were measured on samples of NPL-based ribbons and nanosheets in both solution and in films. The measurements were performed by using an excitation of 360 nm and a calibrated integrating sphere with step increments of 1 nm and an integration time of 0.2 s per data point for three repeated measurements. Light absorption due to scattering inside the sphere was considered for the PLQY measurement by collecting three different spectra: (1) directly exciting the sample in the sphere, (2) indirectly exciting the sample in the sphere, and (3) without the sample in the sphere. For the solution sample, a quartz cuvette with toluene was considered as the reference. For the film, a bare Si substrate was taken as the reference.

### Computational Analysis

The NPLs were modeled with approximately the same shape and aspect ratio as those in the experiments. An individual NPL consists of 1308 beads of unitary mass *m* and diameter σ, which is also taken as our unit of length in simulations. This is illustrated from different perspectives in Figure S10. The length, width, and thickness of the NPLs are 20.3, 7.1, and 2.2 σ, respectively. The effect of the ligands covering the surface of the NPLs is mimicked in a coarse-grained fashion by letting the beads of different NPLs to interact via an attractive 48-24 Lennard-Jones potential,
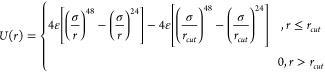
1where ε sets the energy scale and *r*_*cut*_ is the cutoff distance at which the potential is cut and shifted to 0. Here, we set *r*_*cut*_ = 1.3σ. In this way, we implicitly assume a uniform density of ligands on the surface of the NPLs.

To study the formation of ribbons, we place 75 NPLs with random positions and orientations in a cubic box with periodic boundary conditions at a number density ρσ_*L*_^3^ ≈ 3 with σ_*L*_ = 20.3σ the length of an NPL. The assembly is carried out at a reduced temperature , with *k*_*B*_ the Boltzmann’s constant and *T* the temperature, for at least *t* = 1.0 × 10^8^ δ*t*, where δ*t* = 0.002 τ is the simulation time step with τ =  the time unit. A slight change in the values of ρ and T* does not qualitatively alter the results of the simulations. The 3D assembly is studied from nine independent simulations with different initial configurations.

For the analysis in quasi two-dimensional conditions, we initialize the system by randomly placing in the upper part of an elongated box preformed ribbons of different sizes. More specifically, we add two ribbons consisting of 15 NPLs, two ribbons consisting of 10 NPLs, and three ribbons consisting of 6 NPLs. The box has a side of 80σ in the *x* and *y* directions, and a height of 500σ in the *z* direction. We employ periodic boundary conditions in the *x* and *y* directions. The ribbons were first deposited on a substrate using a gravity-like force and subsequently they are let to assemble for *t* = 1.0 × 10^8^ δ*t* at a temperature T* = 7.4. All simulations were performed with LAMMPS.^51^

## Supporting Information Available

Additional TEM images, EDS analysis performed after heating. PL spectra collected at different temperatures. Data on average decay lifetime, PLQY, and radiative and nonradiative recombination rates. Sketch illustrating ligand detachment/relocation and merging of NPLs. Details on the NPLs and ligands used in the simulations (PDF)
Movie S1: Movie recorded from region R1 in BF-TEM imaging mode by using the Ceta camera with a magnification of 6300× and displayed after frame alignment. The real duration of the movie (in 1 fps) is 7.7 minutes. (MP4)
Movie S2: Movie recorded from region R2 in BF-TEM imaging mode by using the Ceta camera with a magnification of 6300× and displayed after frame alignment. The real duration of the movie (in 1 fps) is 9 minutes, as indicated in the time stamp embedded in the movie. (MP4)
Movie S3: Magnified snapshots extracted from Movie S2 display the changes observed in the R2 region where the transformation occurs. (MP4)

## References

[ref1] Zhou Y.; Chen J.; Bakr O. M.; Mohammed O. F. Metal Halide Perovskites for X-ray Imaging Scintillators and Detectors. ACS Energy Letters 2021, 6 (2), 739–768. 10.1021/acsenergylett.0c02430.

[ref2] Wang J.-X.; Wang X.; Yin J.; Gutiérrez-Arzaluz L.; He T.; Chen C.; Han Y.; Zhang Y.; Bakr O. M.; Eddaoudi M.; Mohammed O. F. Perovskite-Nanosheet Sensitizer for Highly Efficient Organic X-ray Imaging Scintillator. ACS Energy Letters 2022, 7 (1), 10–16. 10.1021/acsenergylett.1c02173.

[ref3] Liu J.; Li X.; Wang H.; Yuan G.; Suvorova A.; Gain S.; Ren Y.; Lei W. Ultrathin High-Quality SnTe Nanoplates for Fabricating Flexible Near-Infrared Photodetectors. ACS Appl. Mater. Interfaces 2020, 12 (28), 31810–31822. 10.1021/acsami.0c07847. 32585086

[ref4] Zheng Z.; Wang X.; Shen Y.; Luo Z.; Li L.; Gan L.; Ma Y.; Li H.; Pan A.; Zhai T. Space-Confined Synthesis of 2D All-Inorganic CsPbI_3_ Perovskite Nanosheets for Multiphoton-Pumped Lasing. Advanced Optical Materials 2018, 6 (22), 1800879 10.1002/adom.201800879.

[ref5] Feng J.; Wang J.; Fieramosca A.; Bao R.; Zhao J.; Su R.; Peng Y.; Liew T. C. H.; Sanvitto D.; Xiong Q. All-optical switching based on interacting exciton polaritons in self-assembled perovskite microwires. Science Advances 2021, 7 (46), eabj6627 10.1126/sciadv.abj6627. 34757800 PMC8580323

[ref6] Polavarapu L.; Nickel B.; Feldmann J.; Urban A. S. Advances in Quantum-Confined Perovskite Nanocrystals for Optoelectronics. Adv. Energy Mater. 2017, 7 (16), 1700267 10.1002/aenm.201700267.

[ref7] Zhang B.; Altamura D.; Caliandro R.; Giannini C.; Peng L.; De Trizio L.; Manna L. Stable CsPbBr_3_ Nanoclusters Feature a Disk-like Shape and a Distorted Orthorhombic Structure. J. Am. Chem. Soc. 2022, 144 (11), 5059–5066. 10.1021/jacs.1c13544. 35258285 PMC8949727

[ref8] Zhao J.; Cao S.; Li Z.; Ma N. Amino Acid-Mediated Synthesis of CsPbBr_3_ Perovskite Nanoplatelets with Tunable Thickness and Optical Properties. Chem. Mater. 2018, 30 (19), 6737–6743. 10.1021/acs.chemmater.8b02396.

[ref9] Paul S.; Acharya S. Postsynthesis Transformation of Halide Perovskite Nanocrystals. ACS Energy Letters 2022, 7 (6), 2136–2155. 10.1021/acsenergylett.2c00888.

[ref10] DuBose J. T.; Christy A.; Chakkamalayath J.; Kamat P. V. Transformation of Perovskite Nanoplatelets to Large Nanostructures Driven by Solvent Polarity. ACS Materials Letters 2022, 4 (1), 93–101. 10.1021/acsmaterialslett.1c00663.

[ref11] Li J.; Yuan X.; Jing P.; Li J.; Wei M.; Hua J.; Zhao J.; Tian L. Temperature-dependent photoluminescence of inorganic perovskite nanocrystal films. RSC Adv. 2016, 6 (82), 78311–78316. 10.1039/C6RA17008K.

[ref12] Dang Z.; Dhanabalan B.; Castelli A.; Dhall R.; Bustillo K. C.; Marchelli D.; Spirito D.; Petralanda U.; Shamsi J.; Manna L.; Krahne R.; Arciniegas M. P. Temperature-Driven Transformation of CsPbBr_3_ Nanoplatelets into Mosaic Nanotiles in Solution through Self-Assembly. Nano Lett. 2020, 20 (3), 1808–1818. 10.1021/acs.nanolett.9b05036. 31991086 PMC7997623

[ref13] Otero-Martínez C.; García-Lojo D.; Pastoriza-Santos I.; Pérez-Juste J.; Polavarapu L. Dimensionality Control of Inorganic and Hybrid Perovskite Nanocrystals by Reaction Temperature: From No-Confinement to 3D and 1D Quantum Confinement. Angew. Chem., Int. Ed. 2021, 60 (51), 26677–26684. 10.1002/anie.202109308. PMC9299153 34606151

[ref14] Wang Y.; Li X.; Sreejith S.; Cao F.; Wang Z.; Stuparu M. C.; Zeng H.; Sun H. Photon Driven Transformation of Cesium Lead Halide Perovskites from Few-Monolayer Nanoplatelets to Bulk Phase. Adv. Mater. 2016, 28 (48), 10637–10643. 10.1002/adma.201604110. 27714913

[ref15] Shamsi J.; Rastogi P.; Caligiuri V.; Abdelhady A. L.; Spirito D.; Manna L.; Krahne R. Bright-Emitting Perovskite Films by Large-Scale Synthesis and Photoinduced Solid-State Transformation of CsPbBr_3_ Nanoplatelets. ACS Nano 2017, 11 (10), 10206–10213. 10.1021/acsnano.7b04761. 28945960

[ref16] Pradhan B.; Mushtaq A.; Roy D.; Sain S.; Das B.; Ghorai U. K.; Pal S. K.; Acharya S. Postsynthesis Spontaneous Coalescence of Mixed-Halide Perovskite Nanocubes into Phase-Stable Single-Crystalline Uniform Luminescent Nanowires. J. Phys. Chem. Lett. 2019, 10 (8), 1805–1812. 10.1021/acs.jpclett.9b00832. 30929427

[ref17] Morrell M. V.; He X.; Luo G.; Thind A. S.; White T. A.; Hachtel J. A.; Borisevich A. Y.; Idrobo J.-C.; Mishra R.; Xing Y. Significantly Enhanced Emission Stability of CsPbBr_3_ Nanocrystals via Chemically Induced Fusion Growth for Optoelectronic Devices. ACS Applied Nano Materials 2018, 1 (11), 6091–6098. 10.1021/acsanm.8b01298.

[ref18] Thind A. S.; Luo G.; Hachtel J. A.; Morrell M. V.; Cho S. B.; Borisevich A. Y.; Idrobo J.-C.; Xing Y.; Mishra R. Atomic Structure and Electrical Activity of Grain Boundaries and Ruddlesden–Popper Faults in Cesium Lead Bromide Perovskite. Adv. Mater. 2019, 31 (4), 1805047 10.1002/adma.201805047. 30506822

[ref19] Gomez L.; Lin J.; de Weerd C.; Poirier L.; Boehme S. C.; von Hauff E.; Fujiwara Y.; Suenaga K.; Gregorkiewicz T. Extraordinary Interfacial Stitching between Single All-Inorganic Perovskite Nanocrystals. ACS Appl. Mater. Interfaces 2018, 10 (6), 5984–5991. 10.1021/acsami.7b17432. 29355301 PMC5814954

[ref20] Dey A.; Ye J.; De A.; Debroye E.; Ha S. K.; Bladt E.; Kshirsagar A. S.; Wang Z.; Yin J.; Wang Y.; Quan L. N.; Yan F.; Gao M.; Li X.; Shamsi J.; Debnath T.; Cao M.; Scheel M. A.; Kumar S.; Steele J. A.; Gerhard M.; Chouhan L.; Xu K.; Wu X.-g.; Li Y.; Zhang Y.; Dutta A.; Han C.; Vincon I.; Rogach A. L.; Nag A.; Samanta A.; Korgel B. A.; Shih C.-J.; Gamelin D. R.; Son D. H.; Zeng H.; Zhong H.; Sun H.; Demir H. V.; Scheblykin I. G.; Mora-Seró I.; Stolarczyk J. K.; Zhang J. Z.; Feldmann J.; Hofkens J.; Luther J. M.; Pérez-Prieto J.; Li L.; Manna L.; Bodnarchuk M. I.; Kovalenko M. V.; Roeffaers M. B. J.; Pradhan N.; Mohammed O. F.; Bakr O. M.; Yang P.; Müller-Buschbaum P.; Kamat P. V.; Bao Q.; Zhang Q.; Krahne R.; Galian R. E.; Stranks S. D.; Bals S.; Biju V.; Tisdale W. A.; Yan Y.; Hoye R. L. Z.; Polavarapu L. State of the Art and Prospects for Halide Perovskite Nanocrystals. ACS Nano 2021, 15 (7), 10775–10981. 10.1021/acsnano.0c08903. 34137264 PMC8482768

[ref21] Bhaumik S. Oriented Attachment of Perovskite Cesium Lead Bromide Nanocrystals. ChemistrySelect 2019, 4 (15), 4538–4543. 10.1002/slct.201900142.

[ref22] Otero-Martínez C.; Ye J.; Sung J.; Pastoriza-Santos I.; Pérez-Juste J.; Xia Z.; Rao A.; Hoye R. L. Z.; Polavarapu L. Colloidal Metal-Halide Perovskite Nanoplatelets: Thickness-Controlled Synthesis, Properties, and Application in Light-Emitting Diodes. Adv. Mater. 2022, 34 (10), 2107105 10.1002/adma.202107105. 34775643

[ref23] Sen A.; Chatterjee S.; Sen P. UV-Assisted Conversion of 2D Ruddlesden–Popper Iodide Perovskite Nanoplates into Stable 3D MAPbI_3_ Nanorods. J. Phys. Chem. C 2022, 126 (42), 18057–18066. 10.1021/acs.jpcc.2c05987.

[ref24] Krajewska C. J.; Kaplan A. E. K.; Kick M.; Berkinsky D. B.; Zhu H.; Sverko T.; Van Voorhis T.; Bawendi M. G. Controlled Assembly and Anomalous Thermal Expansion of Ultrathin Cesium Lead Bromide Nanoplatelets. Nano Lett. 2023, 23, 2148 10.1021/acs.nanolett.2c04526. 36884029

[ref25] Xiao X.; Li Y.; Xie R.-J. Blue-emitting and self-assembled thinner perovskite CsPbBr_3_ nanoplates: synthesis and formation mechanism. Nanoscale 2020, 12 (16), 9231–9239. 10.1039/C9NR10885H. 32307479

[ref26] Ye X.; Chen J.; Engel M.; Millan J. A.; Li W.; Qi L.; Xing G.; Collins J. E.; Kagan C. R.; Li J.; Glotzer S. C.; Murray C. B. Competition of shape and interaction patchiness for self-assembling nanoplates. Nat. Chem. 2013, 5 (6), 466–473. 10.1038/nchem.1651. 23695627

[ref27] Petersen N.; Girard M.; Riedinger A.; Valsson O. The Crucial Role of Solvation Forces in the Steric Stabilization of Nanoplatelets. Nano Lett. 2022, 22 (24), 9847–9853. 10.1021/acs.nanolett.2c02848. 36493312 PMC9801426

[ref28] Bekenstein Y.; Koscher B. A.; Eaton S. W.; Yang P.; Alivisatos A. P. Highly Luminescent Colloidal Nanoplates of Perovskite Cesium Lead Halide and Their Oriented Assemblies. J. Am. Chem. Soc. 2015, 137 (51), 16008–16011. 10.1021/jacs.5b11199. 26669631

[ref29] Hudait B.; Dutta S. K.; Patra A.; Nasipuri D.; Pradhan N. Facets Directed Connecting Perovskite Nanocrystals. J. Am. Chem. Soc. 2020, 142 (15), 7207–7217. 10.1021/jacs.0c02168. 32207966

[ref30] Hewavitharana I. K.; Brock S. L. When Ligand Exchange Leads to Ion Exchange: Nanocrystal Facets Dictate the Outcome. ACS Nano 2017, 11 (11), 11217–11224. 10.1021/acsnano.7b05534. 29035564

[ref31] Seyhan M.; Kucharczyk W.; Yarar U. E.; Rickard K.; Rende D.; Baysal N.; Bucak S.; Ozisik R. Interfacial surfactant competition and its impact on poly(ethylene oxide)/Au and poly(ethylene oxide)/Ag nanocomposite properties. Nanotechnol Sci. Appl. 2017, 10, 69–77. 10.2147/NSA.S129468. 28461744 PMC5404797

[ref32] Zhang C.; Fernando J. F. S.; Firestein K. L.; von Treifeldt J. E.; Siriwardena D.; Fang X.; Golberg D. Thermal stability of CsPbBr_3_ perovskite as revealed by in situ transmission electron microscopy. APL Mater. 2019, 7, 071110. 10.1063/1.5108849

[ref33] De Roo J.; Ibáñez M.; Geiregat P.; Nedelcu G.; Walravens W.; Maes J.; Martins J. C.; Van Driessche I.; Kovalenko M. V.; Hens Z. Highly Dynamic Ligand Binding and Light Absorption Coefficient of Cesium Lead Bromide Perovskite Nanocrystals. ACS Nano 2016, 10 (2), 2071–2081. 10.1021/acsnano.5b06295. 26786064

[ref34] Fiuza-Maneiro N.; Sun K.; López-Fernández I.; Gómez-Graña S.; Müller-Buschbaum P.; Polavarapu L. Ligand Chemistry of Inorganic Lead Halide Perovskite Nanocrystals. ACS Energy Letters 2023, 8 (2), 1152–1191. 10.1021/acsenergylett.2c02363.

[ref35] Palazon F.; Di Stasio F.; Lauciello S.; Krahne R.; Prato M.; Manna L. Evolution of CsPbBr_3_ nanocrystals upon post-synthesis annealing under an inert atmosphere. Journal of Materials Chemistry C 2016, 4 (39), 9179–9182. 10.1039/C6TC03342C.

[ref36] Hristu R.; Stanciu S. G.; Tranca D. E.; Stanciu G. A. Electron beam influence on the carbon contamination of electron irradiated hydroxyapatite thin films. Appl. Surf. Sci. 2015, 346, 342–347. 10.1016/j.apsusc.2015.03.214.

[ref37] Palazon F.; Akkerman Q. A.; Prato M.; Manna L. X-ray Lithography on Perovskite Nanocrystals Films: From Patterning with Anion-Exchange Reactions to Enhanced Stability in Air and Water. ACS Nano 2016, 10 (1), 1224–1230. 10.1021/acsnano.5b06536. 26617344 PMC4734608

[ref38] Boles M. A.; Engel M.; Talapin D. V. Self-Assembly of Colloidal Nanocrystals: From Intricate Structures to Functional Materials. Chem. Rev. 2016, 116 (18), 11220–11289. 10.1021/acs.chemrev.6b00196. 27552640

[ref39] Toso S.; Baranov D.; Giannini C.; Manna L. Structure and Surface Passivation of Ultrathin Cesium Lead Halide Nanoplatelets Revealed by Multilayer Diffraction. ACS Nano 2021, 15 (12), 20341–20352. 10.1021/acsnano.1c08636. 34843227 PMC8717630

[ref40] Lin D.; Li Y. Large-Scale 2D-Confined Self-Assembly of Colloidal Nanoparticles via Dynamic Ice Crystal Templates. ACS Central Science 2022, 8 (5), 510–512. 10.1021/acscentsci.2c00531. 35647278 PMC9136964

[ref41] Arciniegas M. P.; Stasio F. D.; Li H.; Altamura D.; De Trizio L.; Prato M.; Scarpellini A.; Moreels I.; Krahne R.; Manna L. Self-Assembled Dense Colloidal Cu_2_Te Nanodisk Networks in P3HT Thin Films with Enhanced Photocurrent. Adv. Funct. Mater. 2016, 26 (25), 4535–4542. 10.1002/adfm.201600751.

[ref42] Lin C.-K.; Zhao Q.; Zhang Y.; Cestellos-Blanco S.; Kong Q.; Lai M.; Kang J.; Yang P. Two-Step Patterning of Scalable All-Inorganic Halide Perovskite Arrays. ACS Nano 2020, 14 (3), 3500–3508. 10.1021/acsnano.9b09685. 32057230

[ref43] Fan Z.; Grünwald M. Orientational Order in Self-Assembled Nanocrystal Superlattices. J. Am. Chem. Soc. 2019, 141 (5), 1980–1988. 10.1021/jacs.8b10752. 30628775

[ref44] Noh S. H.; Jeong W.; Lee K. H.; Yang H. S.; Suh E. H.; Jung J.; Park S. C.; Lee D.; Jung I. H.; Jeong Y. J.; Jang J., Photocrosslinkable Zwitterionic Ligands for Perovskite Nanocrystals: Self-Assembly and High-Resolution Direct Patterning. Adv. Functional Mater. 2023, 2304004. 10.1002/adfm.202304004

[ref45] Mandal A.; Ghosh A.; Senanayak S. P.; Friend R. H.; Bhattacharyya S. Thickness-Attuned CsPbBr_3_ Nanosheets with Enhanced p-Type Field Effect Mobility. J. Phys. Chem. Lett. 2021, 12 (5), 1560–1566. 10.1021/acs.jpclett.0c03815. 33534600

[ref46] Dou L.; Wong A. B.; Yu Y.; Lai M.; Kornienko N.; Eaton S. W.; Fu A.; Bischak C. G.; Ma J.; Ding T.; Ginsberg N. S.; Wang L.-W.; Alivisatos A. P.; Yang P. Atomically thin two-dimensional organic-inorganic hybrid perovskites. Science 2015, 349 (6255), 1518–1521. 10.1126/science.aac7660. 26404831

[ref47] Ha S. K.; Mauck C. M.; Tisdale W. A. Toward Stable Deep-Blue Luminescent Colloidal Lead Halide Perovskite Nanoplatelets: Systematic Photostability Investigation. Chem. Mater. 2019, 31 (7), 2486–2496. 10.1021/acs.chemmater.8b05310.

[ref48] Vonk S. J. W.; Fridriksson M. B.; Hinterding S. O. M.; Mangnus M. J. J.; van Swieten T. P.; Grozema F. C.; Rabouw F. T.; van der Stam W. Trapping and Detrapping in Colloidal Perovskite Nanoplatelets: Elucidation and Prevention of Nonradiative Processes through Chemical Treatment. J. Phys. Chem. C 2020, 124 (14), 8047–8054. 10.1021/acs.jpcc.0c02287. PMC7217613 32421082

[ref49] Di Stasio F.; Imran M.; Akkerman Q. A.; Prato M.; Manna L.; Krahne R. Reversible Concentration-Dependent Photoluminescence Quenching and Change of Emission Color in CsPbBr_3_ Nanowires and Nanoplatelets. J. Phys. Chem. Lett. 2017, 8 (12), 2725–2729. 10.1021/acs.jpclett.7b01305. 28581755

[ref50] Kilaas R. Optimal and near-optimal filters in high-resolution electron microscopy. J. Microsc. 1998, 190 (1–2), 45–51. 10.1046/j.1365-2818.1998.3070861.x.

[ref51] Thompson A. P.; Aktulga H. M.; Berger R.; Bolintineanu D. S.; Brown W. M.; Crozier P. S.; in ’t Veld P. J.; Kohlmeyer A.; Moore S. G.; Nguyen T. D.; Shan R.; Stevens M. J.; Tranchida J.; Trott C.; Plimpton S. J. LAMMPS - a flexible simulation tool for particle-based materials modeling at the atomic, meso, and continuum scales. Comput. Phys. Commun. 2022, 271, 108171 10.1016/j.cpc.2021.108171.

