# Supplementary material for: Real-Time In Situ Observation of CsPbBr3 Perovskite Nanoplatelets Transforming into Nanosheets
Source: ACS Nano. 2023 Jul 5;17(14):13648–58. doi: 10.1021/acsnano.3c02477 (PMC10373526; doi:10.1021/acsnano.3c02477)
Supplement: Supplementary file 1 — nn3c02477_si_001.pdf [file nn3c02477_si_001.pdf]

Supporting Information for

Real-time In situ Observation of $CsPbBr_3$ Perovskite Nanoplatelets Transforming into Nanosheets

Aarya Prabhakaran,[ǂ§] Zhiya Dang,[£] Rohan Dhall[ʗ], Fabrizio Camerin[¥], Susana Marín-Aguilar[¥], Balaji Dhanabalan,[ǂʅ] Andrea Castelli,[ǂʅ] Rosaria Brescia[ǂ], Liberato Manna[ǂ], Marjolein Dijkstra[¥], and Milena P. Arciniegas[ǂ]*

[ǂ]Istituto Italiano di Tecnologia. Via Morego 30, 16163. Genoa, Italy.

[§]Dipartimento di Chimica e Chimica Industriale, Università degli Studi di Genova, Via Dodecaneso, 31, 16146, Genova, Italy

[ʗ]National Center for Electron Microscopy, Molecular Foundry, Lawrence Berkeley National Laboratory, Berkeley, California 94720, United States.

[£]School of Materials. Shenzhen Campus of Sun Yat-sen University, No. 66, Gongchang Road, Guangming District, Shenzhen, Guangdong 518107, P.R. China.

[¥]Soft Condensed Matter, Debye Institute for Nanomaterials Science, Utrecht University, Princetonplein 1, 3584CC, Utrecht, The Netherlands.

Correspondence should be addressed to milena.arciniegas@iit.it.

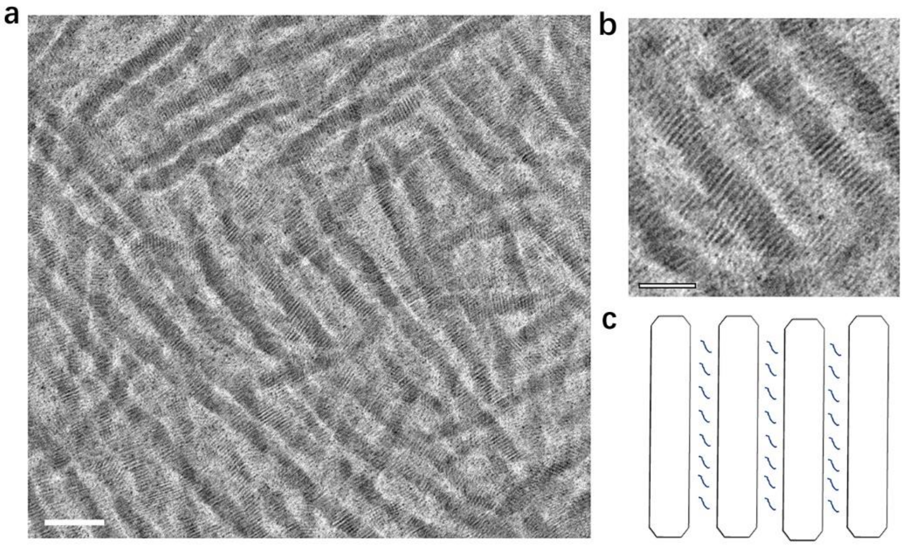

Figure S1. a, Representative bright-field TEM image of a highly concentrated area of ribbons in the sample. Scale bar: 100 nm **b,** Magnified view of a zone containing a group of parallel ribbons. The closer distance between parallel ribbons is ca. 3 nm. Scale bar: 20 nm. **c,** Sketch illustrating a top view of a short ribbon formed by 4 self-aligned NPLs connected through the ligand shell.

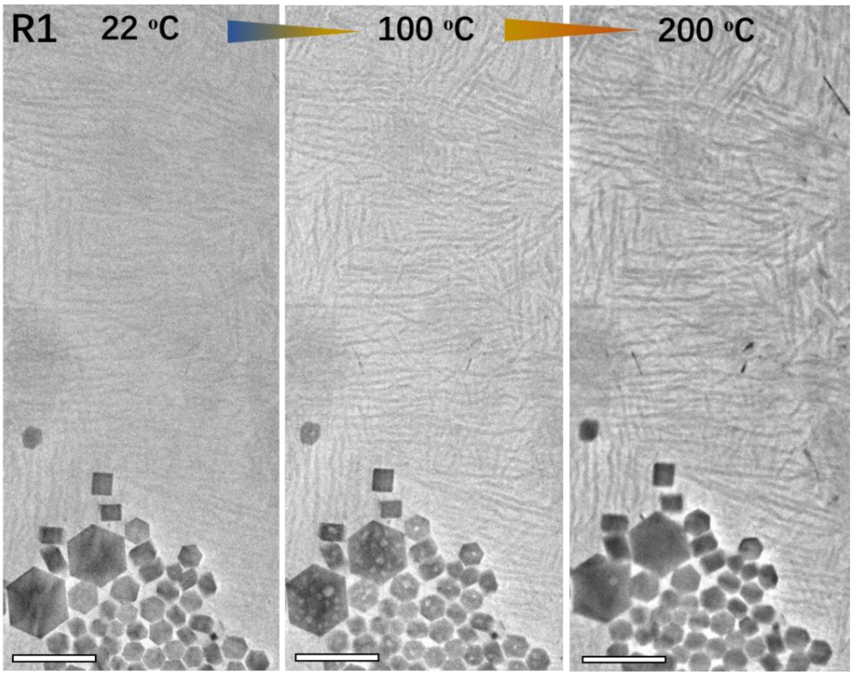

Figure S2. Bright-field TEM images of a region containing a low concentration of ribbons (R1) collected at different temperatures. Scale bars: 500 nm. The number of ribbons is ca. 40 per 500 x 500 nm^2.

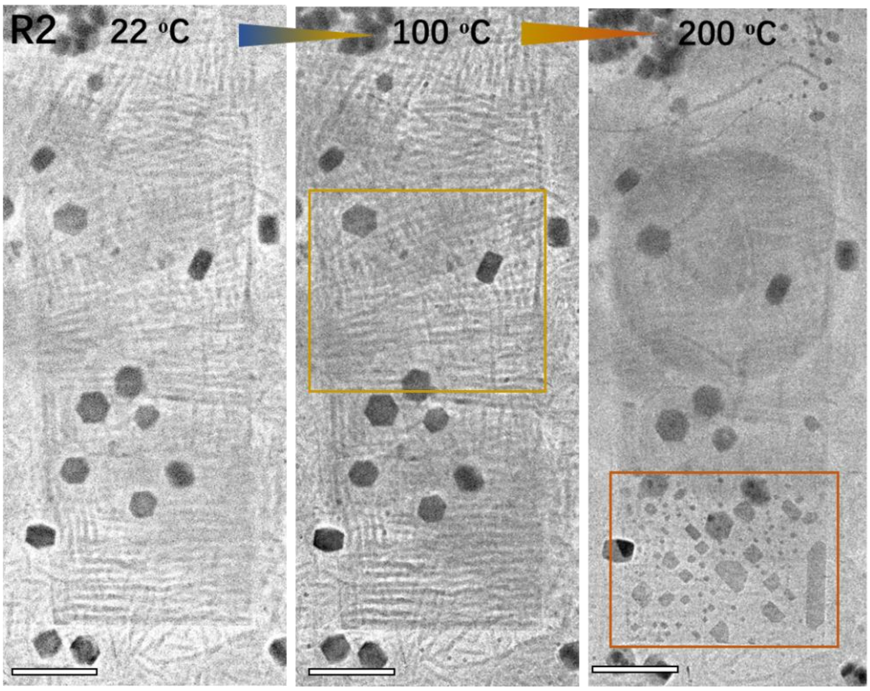

Figure S3. Bright-field TEM images of a region containing a high concentration of ribbons (R2) collected at different temperatures. Scale bars: 500 nm. The number of ribbons is ca. 60 per 500 x 500 nm^2. The rectangles highlight the areas where further morphological details were obtained.

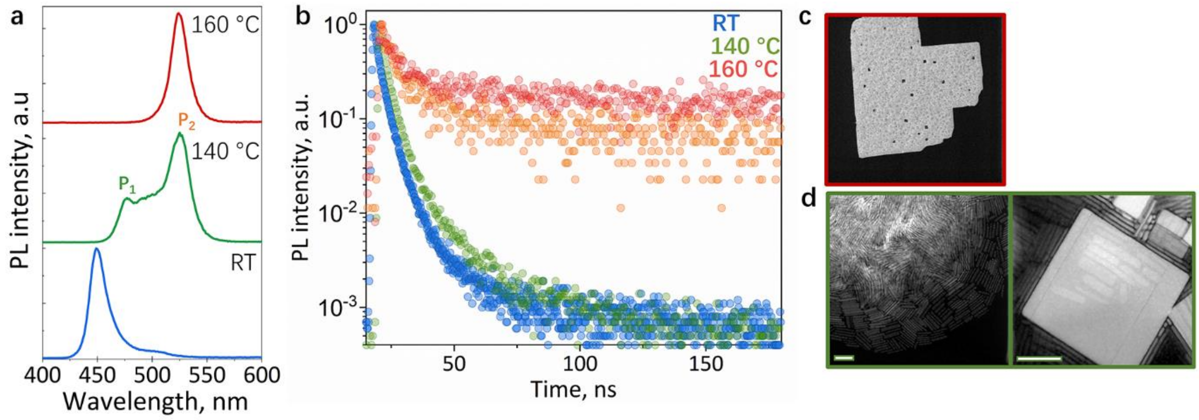

Figure S4. a-b, PL spectra (in a) and time-resolved PL decays (in b) collected from a batch of samples dispersed in toluene and heated at different temperatures, from 22 °C (RT) to 160 °C. The decay lifetime of the high (P_1) and low (P_2) energy peaks observed from the solution heated at 140 °C is displayed in light green and light orange in panel b. **c-d,** High-angle annular dark field (HAADF) scanning TEM (STEM) images of the objects produced after heating at 160 °C (in c) and at 140 °C (in d). Scale bars: 50 nm.

Figure S4 shows a collection of PL spectra from different temperatures, which indicates that the NPLs observed at room temperature undergo a full transformation at 160 °C. At 140 °C, we observe different emission peaks, which originated from objects with different morphology, as observed in the STEM images in Figure S4d. At 160 °C, only large nanosheets were observed (Figure S4c).

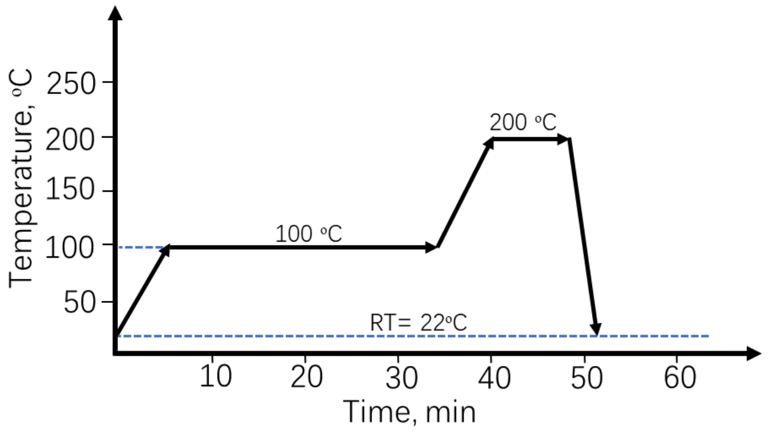

Figure S5. Heating ramps established for the in-situ TEM experiment starting from a room temperature (RT) of ca. 22 °C up to 200 °C.

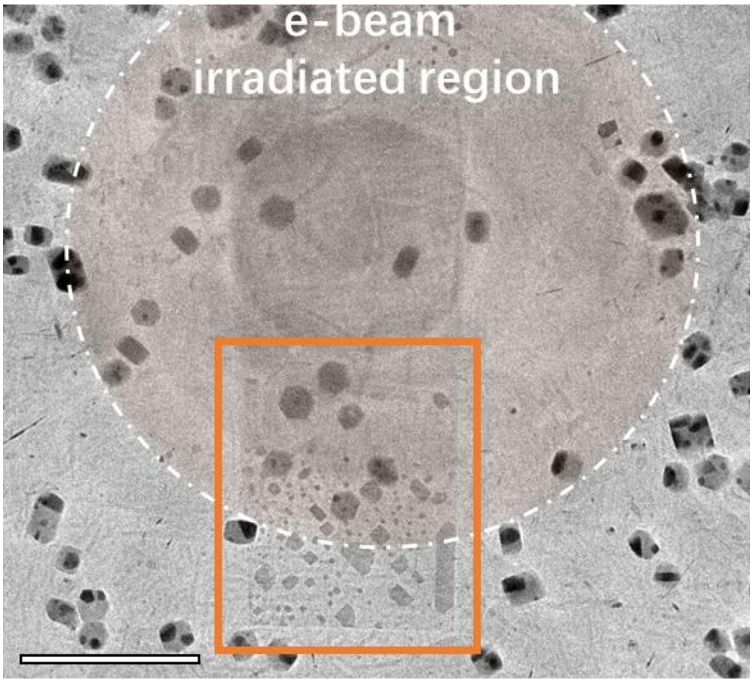

Figure S6. Bright-field TEM image collected at 200 °C showing regions from the R2 area impinged previously with different electron doses: the darkest circle with the highest dose, exhibiting the formation of cross-linked carbon, and the lightest ones with the lowest dose. The orange rectangle frames the region in which significant morphological changes are observed at high temperature. Scale bar: 1 µm.

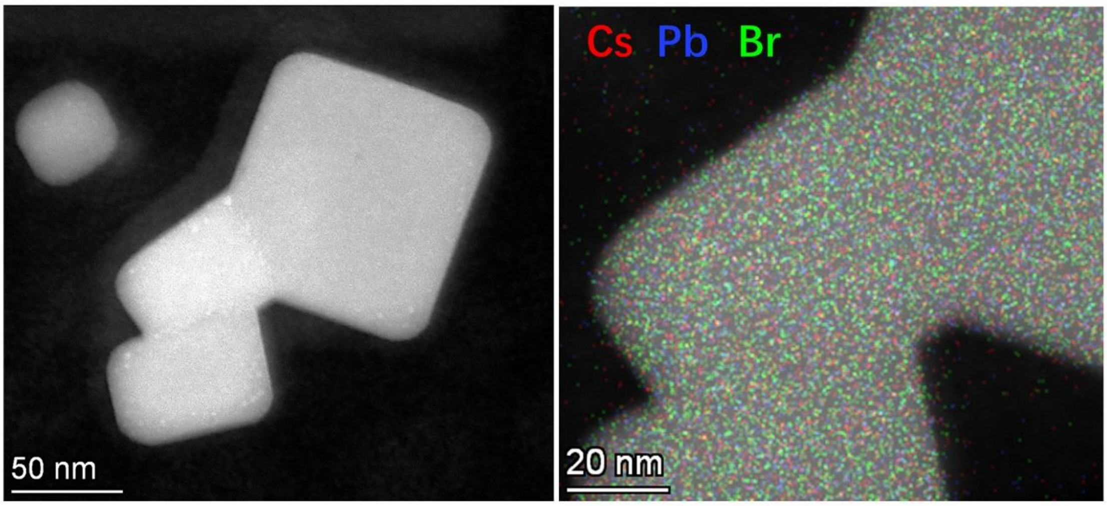

Figure S7. HAADF-STEM image (left) and the corresponding STEM-EDS elemental mapping (right) for Cs, Pb, and Br of a representative structure observed after the heating experiments confirming the chemical composition of $CsPbBr_3$ nanosheets produced by the merging of small neighboring nanosheets.

Table S1. Elemental mapping analysis for Cs, Pb, and Br, obtained via STEM-EDS performed on samples after the in-situ TEM heating experiments. According to this quantification collected from 4 different regions, the resulting Cs:Pb:Br average ratio in atomic % is 1:1:3. Fit error <3%.

Element	Family	Atomic fraction, %	Mass fraction
Br	K	5.87	4.06
Cs	L	2.29	2.63
Pb	L	1.84	3.31

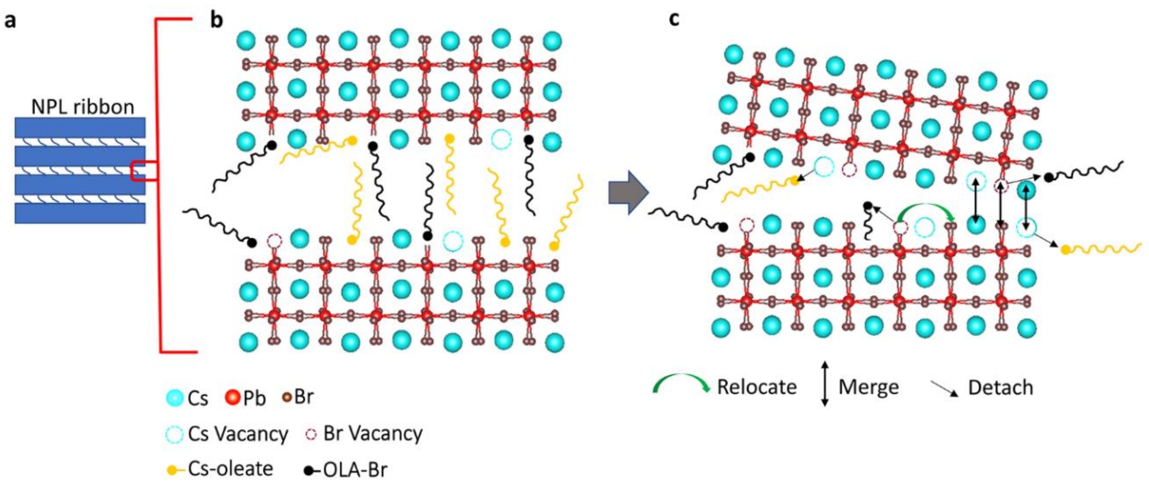

Figure S8. a-c, Sketches showing the detachment and relocation of ligands between two neighboring NPLs within a ribbon (in a). The surface of the initial NPLs is formed by vacancies, bound ligands, and surfaces sites without ligands (in b).[1-2] In this scenario, Cs-oleate and oleylammonium-Br ligand pairs can detach fully from the surface leaving Cs and Br vacancies and/or relocate occupying other vacancy sites. This ligand mobility is favored on the portion of the surface near the edges where ligands can rotate/bend with one end attached to the surface and one end out of the lattice, facilitating the merging of the lattices from the portion near the edge (in c).

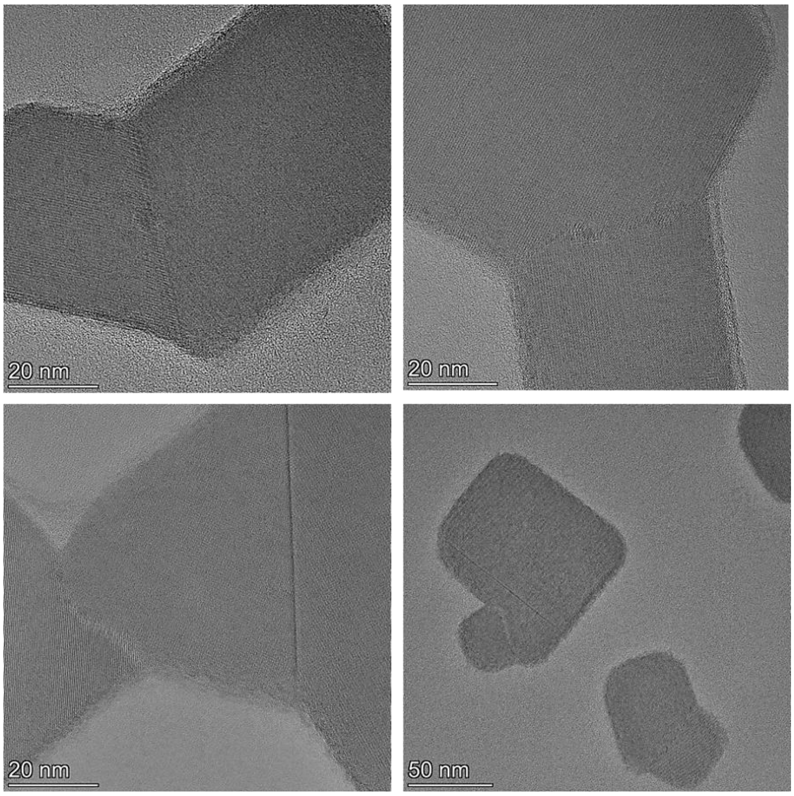

Figure S9. High-resolution TEM images collected from different nanosheets produced after the in-situ heating TEM experiments. These images evidence the formation of imperfect perovskite lattices with grain boundaries and RP-planar faults located at the merging interface of intermediates that instead show a continuous perovskite lattice.

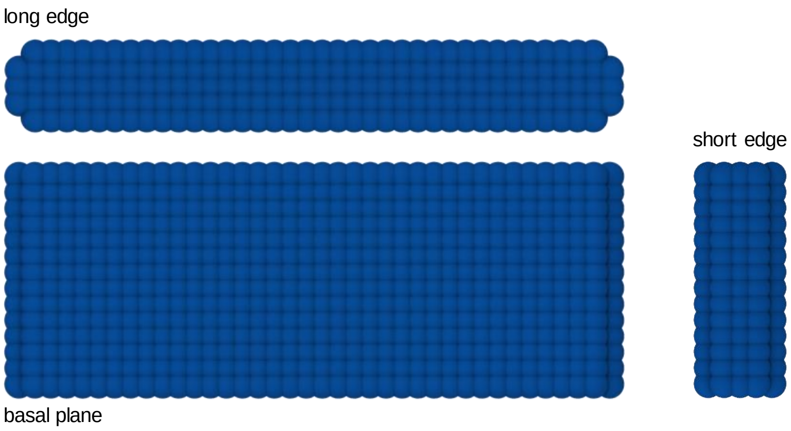

Figure S10. Modeling of individual NPLs. Each NPL is made of 1308 beads and approximately retains the same shape and aspect ratio as in the experiments. The three snapshots report the same NPL from different perspectives, showing the long and short edges, and the basal plane.

Table S2. Fitting parameters of the PL time decay profiles obtained from solution and drop-cast films are presented in Figure S4 and Figure 5 of the main document, respectively. τ_1, τ_2,τ_3, and τ_{avg} are the three-exponential time decay fitting and their average value. In bold, are the τ values that contribute the most to the decay lifetime. In the case of nanosheets, there is a balanced contribution from the slow and fast components. RT: room temperature.

Sample description		τ_1, ns	A_1, %	τ_2, ns	A_2, %	τ_3, ns	A_3, %	τ_{avg}, ns
RT	Solution	1.17	21.90	3.86	69.66	17.26	8.45	8.14
	Drop-cast film	5.06	4.19	**7.9613**	**65.77**	24.51	30.04	17.42
140°C-P_1	Solution	1.62	39.6	5.45	52.47	33.98	7.93	17.94
	Drop-cast film	4.46	20.73	**6.63**	**61.98**	24.4	17.28	14.51
140 °C-P_2	Solution	2.79	9.71	14.21	18.94	157.68	71.35	153.97
	Drop-cast film	**3.89**	**30.06**	**33.21**	**39.13**	**197.23**	**34.16**	**167.93**
160°C	Solution	2.2	0.82	7.24	18.66	160.65	80.52	159.04
	Drop-cast film	**4.09**	**31.25**	**32.41**	**37.74**	**201**	**31.02**	**170.50**

Average PL decay lifetime τ_{avg} was calculated using equation 1 below[3]:

$$\tau_{avg} = \frac{A_1\tau_1^2+A_2\tau_2^2+A_3\tau_3^2}{A_1\tau_1+A_2\tau_2+A_3\tau_3} \qquad \text{Equation 1}$$

With the average PL decay lifetime calculated from the PL decay fitting presented in Table S2 and the PLQY values of the starting NPLs and the transformed structures (nanosheets), we calculated the average radiative and non-radiative rates of the emission by using equations 2 and 3 below[4-5]:

$$\tau_{avg} = \frac{1}{K_r+K_{nr}} \qquad \text{Equation 2}$$

$$PLQY = \frac{K_r}{K_r+K_{nr}} \qquad \text{Equation 3}$$

The calculated radiative (K_r) and non-radiative (K_{nr}) recombination rate constants are presented in Table S3.

Table S3. PLQY values of the initial nanoplates forming ribbon structures and the nanosheets obtained through the heat-induced transformation from drop-cast films and in solution. The table also shows the calculated radiative (K_r) and non-radiative (K_{nr}) recombination rates calculated from the PLQY and τ_{avg} from Table S2 and using equations 2 and 3. RT: room temperature.

Sample description	PLQY	K_r, MHz	K_{nr}, MHz	K_{nr}/K_r
Drop-cast film, RT	23%	13.2	44.2	3.35
Drop-cast film, 160°C	8%	0.47	5.39	11.47
Solution, RT	25%	30.71	92.14	3.00
Solution, 160°C	12%	0.75	5.53	7.37

Movie S1. Movie recorded from region R1 in BF-TEM imaging mode by using the Ceta camera with a magnification of 6300 X and displayed after frame alignment. The real duration of the movie (in 1 fps) is 7.7 minutes.

Movie S2. Movie recorded from region R2 in BF-TEM imaging mode by using the Ceta camera with a magnification of 6300 X and displayed after frame alignment. The real duration of the movie (in 1 fps) is 9 minutes, as indicated in the time stamp embedded in the movie.

Movie S3. Magnified snapshots extracted from Movie S2 display the changes observed in the R2 region where the transformation occurs.

References

1. Toso, S.; Baranov, D.; Giannini, C.; Manna, L., Structure and Surface Passivation of Ultrathin Cesium Lead Halide Nanoplatelets Revealed by Multilayer Diffraction. *ACS Nano* **2021,** *15* (12), 20341-20352.
2. Fiuza-Maneiro, N.; Sun, K.; López-Fernández, I.; Gómez-Graña, S.; Müller-Buschbaum, P.; Polavarapu, L., Ligand Chemistry of Inorganic Lead Halide Perovskite Nanocrystals. *ACS Energy Letters* **2023,** *8* (2), 1152-1191.
3. Sillen, A.; Engelborghs, Y., The Correct Use of "Average" Fluorescence Parameters. *Photochem. Photobiol.* **1998,** *67* (5), 475-486.
4. Omogo, B.; Aldana Jf Fau - Heyes, C. D.; Heyes, C. D., Radiative and Non-Radiative Lifetime Engineering of Quantum Dots in Multiple Solvents by Surface Atom Stoichiometry and Ligands. (1932-7447 (Print)).
5. Lakowicz, J. R., *Principles of Fluorescence Spectroscopy*. Springer New York, NY, 2006.
